# Spatio-temporal six-year retrospective study on dermatophytosis in Rio de Janeiro, Southeast Brazil: A tropical tourist locality tale

**DOI:** 10.1371/journal.pntd.0010865

**Published:** 2023-04-03

**Authors:** Simone Cristina Pereira Brito, Márcia Ribeiro Pinto, Lucas Martins Alcântara, Nathália Faria Reis, Thiago Lacerda Durães, Christina Teresa Machado Bittar, Jeferson Carvalhaes de Oliveira, Elisabeth Martins da Silva da Rocha, Ricardo Luiz Dantas Machado, Ricardo José de Paula Souza e Guimarães, Andréa Regina de Souza Baptista

**Affiliations:** 1 Center for Microorganisms’ Investigation, Biomedical Institute, Department of Microbiology and Parasitology, Fluminense Federal University, Niterói, Rio de Janeiro, Brazil; 2 Laboratory of Biochemistry and Immunology of Mycoses, Biomedical Institute, Department of Microbiology and Parasitology, Fluminense Federal University, Niterói, Rio de Janeiro, Brazil; 3 Bittar Laboratory – Diagnostic Medicine, Niterói, Rio de Janeiro, Brazil; 4 Gamboa Hospital, Santa Casa da Misericórdia do Rio de Janeiro, Rio de Janeiro, Brazil; 5 Geoprocessing Laboratory, Evandro Chagas Institute/SVS/MS, Ananindeua, Pará, Brazil; 6 Rede Micologia RJ —Fundação de Amparo à Pesquisa do Estado do Rio de Janeiro (FAPERJ), Rio de Janeiro, Brazil; University Hospitals Sussex NHS Foundation Trust, UNITED KINGDOM

## Abstract

*Trichophyton*, *Microsporum*, *Nannizzia* and *Epidermophyton* genera cause dermatophytosis, the most common and highly contagious infectious skin disease. Rio de Janeiro is one of the most visited cities in the Southern Hemisphere, located in the most visited state of Brazil. This retrospective study investigated epidemiological and laboratorial aspects of dermatophytosis in Rio de Janeiro state, Brazil, by using spatiotemporal analysis. More than half of all individuals were infected by one or more dermatophytes. A variation between 18 and 106 years-old of the studied population was verified, and women more frequently affected. Patients were more frequently infected by *Trichophyton* spp., most of them *T*. *rubrum*, followed by *T*. *mentagrophytes*. *M*. *canis* and *N*. *gypsea* were more frequently isolated in the age group between 40 and 60 years old, while *T*. *rubrum* predominates among younger patients. All species presented homogeneous distribution while *T*. *tonsurans* appears to be restricted to the Rio de Janeiro capital and *E*. *floccosum* to the municipality of Macaé (190 Km apart from RJ). Rio de Janeiro state presented spatial clusters of dermatophytosis with high density in Guanabara Bay (*E*. *floccosum*, *M*. *canis*, *N*. *gypsea*, *T*. *tonsurans*) and Niterói (*T*. *rubrum*, *T*. *mentagrophytes*) but low density in Macaé (*E*. *floccosum*). Significant spatiotemporal clusters on dermatophytosis cases were detected in distinct municipalities (p-value ≤ 0.05). The Vulnerability Index (r = 0.293) and Demographic Density (r = 0.652) distributed according to neighborhoods in Niterói were direct related with dermatophytosis cases whereas Income (r = -0.306) was inversely correlated (p-value ≤ 0.05). The dermatophytosis spatiotemporal distinct distribution after two major international events in Rio de Janeiro, Brazil, highlight the pressing need for specific measures of its prevention and controlling. This is particularly relevant in touristic tropical localities which must consider both socio-economical and traveler’s medicine variables.

## Author summary

Four genera of fungi cause dermatophytosis, the most common and highly contagious infectious skin disease. Distinguishing dermatophyte species is crucial since ecological origin (geophilic, zoophilic or anthropophilic) provides clues about the source of infection, contributing to avoid reinfection and to the establishment of prevention measures. Rio de Janeiro is one of the most visited cities in the Southern Hemisphere, located in the most visited state of Brazil. This retrospective study investigated epidemiological and laboratorial aspects of dermatophytosis in Rio de Janeiro state, Brazil, by using spatiotemporal analysis. More than half of all individuals were infected by one or more dermatophytes. *Microsporum canis* and *Nannizzia gypsea* were more frequently isolated in the age group between 40 and 60 years old while *T*. *rubrum* predominates among younger patients. All species presented homogeneous distribution while *T*. *tonsurans* appears to be restricted to Rio de Janeiro capital and *Epydermophyton floccosum* to the municipality of Macaé. Significant spatiotemporal clusters on dermatophytosis cases were detected in distinct municipalities (p-value ≤ 0.05). Dermatophytosis spatiotemporal distinct distribution after two major international events in Rio de Janeiro, Brazil (2014 Football World Cup and the 2016 Olympic Games), highlights the pressing need for specific measures of prevention and controlling.

## 1. Introduction

Approximately a billion people are estimated to have skin, nail and hair mycosis, known as dermatomycosis. Altogether, this condition constitutes the 4^th^ leading cause of nonfatal disease burden worldwide. However, research and funding investments still do not turn into improvements for relief of the relative disability of skin diseases [1], therefore considered a public health problem [2]. Dermatophytosis, caused by different septate hyaline filamentous fungi species belonging to four genera: *Trichophyton*, *Microsporum*, *Nannizzia* and *Epidermophyton*, are the most common infectious skin disease and its epidemiology greatly varies. Different countries and regions, ethnic groups, lifestyles, socioeconomic status and climatic zones, fungal characteristics, plus therapeutic options, among other factors, can influence these species’ distribution and overtime changes can be detected [3]. Besides, clinical manifestations depend on the species, anatomical location, and immune status of the patient [4]. In fact, data shows that 20 to 25% of the world’s population is affected by dermatophytosis [5–8] while 10–15% will have at least one episode in their lifetime [9].

Human patients of both genders and of any age can be affected, and although dermatophytes ubiquitous distribution, warm and humid climates such as tropical and subtropical areas are known to contribute with a large number of cases in Latin America, Africa and Asia [6,10,11]. In Brazil, dermatophytosis prevalence varied from 18.2% to 23.2% [12–15].

Rio de Janeiro is the most visited state in Brazil. Rio de Janeiro, Brazil’s former capital and second-largest city is internationally known for one of the seven new wonders of the world—the Christ the Redeemer statue. It is also a city of vibrant nature due to proximity to the sea and the towering cliffs surrounded by forests. Population mobility, changes in human lifestyle, environmental conditions such as humidity and temperature, diagnosis, and advents of antifungal drugs will continuously drive the dermatophyte evolution in the skin microenvironment and consequently within populations [16]. Understanding dermatophytosis behavior in an area and its determinants is essential for implementing interventions aimed at reducing fungal transmission through the identification of areas at high vulnerability. In order to investigate the relationships between living conditions, environmental factors and fungal diseases, it is necessary to adopt geoprocessing techniques which include spatiotemporal analysis [17].

This study is a six-year retrospective investigation by geoprocessing on epidemiological and laboratorial aspects of dermatophytosis in different locations of Rio de Janeiro state, Brazil.

## 2. Methods

### 2.1. Ethics statement

The study was approved by and conducted according to the norms of the Ethics Committee of the Antonio Pedro University Hospital (CEP-HUAP) protocol number n° 3.798.208, January 10th, 2020.

### 2.2. Study design

From January 2014 to December 2020, medical reports of patients with clinical suspicion of superficial and cutaneous mycoses were obtained from a private reference diagnostic laboratory in the city of Niterói, Rio de Janeiro, Southeast region of Brazil (S1 Fig). Among those, individuals from the Rio de Janeiro Metropolitana Region, (Health State Secretary of Rio de Janeiro—SES/RJ, 2022), aged 18 years old or above, regardless of gender or ethnicity, diagnosed as infected by at least one dermatophyte, were included. Clinical-epidemiological and laboratorial data such as the isolated fungal agent, the clinical suspicion, the anatomical site of the biological sample, the patient’s age and gender and area of residence were registered on a standardized data collection form.

### 2.3. Study area

The study was carried out in the state of Rio de Janeiro and the municipality of Niterói was chosen to further perform spatiotemporal analysis of dermatophytosis.

According to the Brazilian Institute of Geography and the Statistics (IBGE), Rio de Janeiro state occupies an area of 43,750.425 km^2^, an estimated population in 2021 of almost 18 million inhabitants, with a demographic density of approximately 366 inhabitants/km^2^. Niterói borders Rio de Janeiro capital (13 km apart) and belongs to the Metropolitana Region (Health State Secretary of Rio de Janeiro—SES/RJ, 2022), with a 133.757 km^2^ territorial area, an estimated population in 2021 of almost 520.000 inhabitants and a ten times higher demographic density, of around 3,700 inhabitants/km^2^. This municipality has the seventh highest human development index (IDH) in the country and the first in the state [18].

The Brazilian airports Tom Jobim International and Santos Dumont Airport are located in Rio de Janeiro capital, and represent two of the busiest gates of entrance for international tourists in this country. Indeed, Rio de Janeiro is a worldwide-known first choice for leisure travel and the second for business and international events. Moreover, the Port of Rio de Janeiro is Brazil’s third busiest port in terms of cargo volume, and it is the center for cruise vessels [19]. Niterói lies across Guanabara Bay facing the city of Rio de Janeiro, also receiving a strong touristic influx. This is due to its many beaches, historical architecture and modern buildings such as the Museum of Contemporary Art and also because of its outdoor activities, including the gold medal-winning sport–sailing [20].

### 2.4. Laboratorial analysis

Biological samples were collected based on clinical conditions and type of lesion by physicians from the distinct outpatient clinics from different Rio de Janeiro municipalities. For diagnostic purposes, direct mycological examination (DME) and mycological culture were conducted by the laboratorial technical staff. Samples were collected with scalpel, slides, curette and/or tweezers and submitted to routine mycological examination as follows: DME after KOH 10–40% and seeding onto Sabouraud agar 2% dextrose (BD, Franklin Lakes, NJ, USA) and Mycosel (BD, Franklin Lakes, NJ, USA). All media were incubated at room temperature (25–28 °C) and observed over four weeks for fungal growth. Colonies showing features suggestive of one of the four dermatophytes genus were subcultivated on Sabouraud agar 2% dextrose (BD, Franklin Lakes, NJ, USA) at room temperature for colony isolation. The fungi were identified based on macro- and microscopic characteristics.

### 2.5. Statistical and spatio-temporal analysis

The data were processed and analyzed with the aid of statistical software BioEstat 5.3 (Belém, PA, Brazil). To analyze the results, the Chi-square test and the Fischer Exact Test were used, and the nature of the variables was considered. Laboratory methods performances were compared with McNemar test. For all tests, the level of significance was set at p ≤ 0.05.

The region, federation units, municipalities, districts and neighborhoods boundaries and socioeconomic variables were obtained oh the IBGE [18] (https://www.ibge.gov.br/geociencias/downloads-geociencias.html) and the Regions of Rio de Janeiro boundaries were obtained of the Foundation State Center for Statistics, Research and Training of Public Servants in Rio de Janeiro [21] (http://arquivos.proderj.rj.gov.br/sefaz_ceperj_imagens/Arquivos_Ceperj/ceep/informacoes-do-territorio/cartografia-fluminense/Divis%C3%A3o%20municipal%20e%20regional%20fluminense%20-%202018%20-%20CEPERJ.pdf). Brazilian Geodetic System [22] (https://www.sirgas.org/) datum and Geographic Coordinate System were used.

The georeferencing of “cases” was obtained using the neighborhoods, which was derived from the census sectors of the 2010 IBGE Census (https://www.ibge.gov.br/estatisticas/downloads-estatisticas.html).

The socioeconomic variables of the census sectors were chosen for the construction of the vulnerability index (VI): income up to one minimum wage (income) and demographic density. Pearson’s correlation coefficient (r) was performed to verify the association between the positive mycological exams, VI, and the variables (income and demographic density) by using ISwR tools in the R software (https://www.r-project.org/).

The spatial analysis performed were: (1) map of spatial distribution (choropleth maps) to visualize the location of the number of cases in the municipalities and neighborhoods and the vulnerability index in the neighborhoods; (2) Kernel density estimation (KDE) to identify the location of clusters for case occurrences; (3) spatial scanning map (Scan) to identify spatial and temporal clusters with statistical significance.

For the choropleth maps construction the ArcGIS software version 10.4 (https://www.arcgis.com), was used with the parameters "No cases" (white color), "1–5" (dark green), "6–15" (light green), "16–50" (yellow), "51–500" (orange) and "> 500" (red) for spatial distribution of cases; and "0.001–0.125" (Low—green), "0.126–0.250" (Medium—yellow), "0.251–0.500" (High—orange), "0.500–1" (Very High—red) for vulnerability index. KDE was applied in cases of dermatophytosis using the quartic function, density calculation and adaptive radius to evaluate the presence of clusters in the TerraView software (http://www.dpi.inpe.br/terralib5/wiki/doku.php). The Scan was applied to detect spatiotemporal clusters while statistical significance was verified in the SaTScan software (https://www.satscan.org/) using the Discrete Poisson model.

## 3. Results

A total of 7,927 biological samples were received for fungal etiology investigation between 2014 and 2020, including: skin (dermal scales) and nail scrapings, skin biopsies, liquids and fluids (punctures, liquor, urine, bronchoalveolar lavage, peritoneal liquid, sputum, among others). Of these, those with suspected dermatomycoses were screened, totaling around one third of all diagnostic demand of the Bittar Laboratory (n = 2,724; 34.4%).

**Fig 1:**
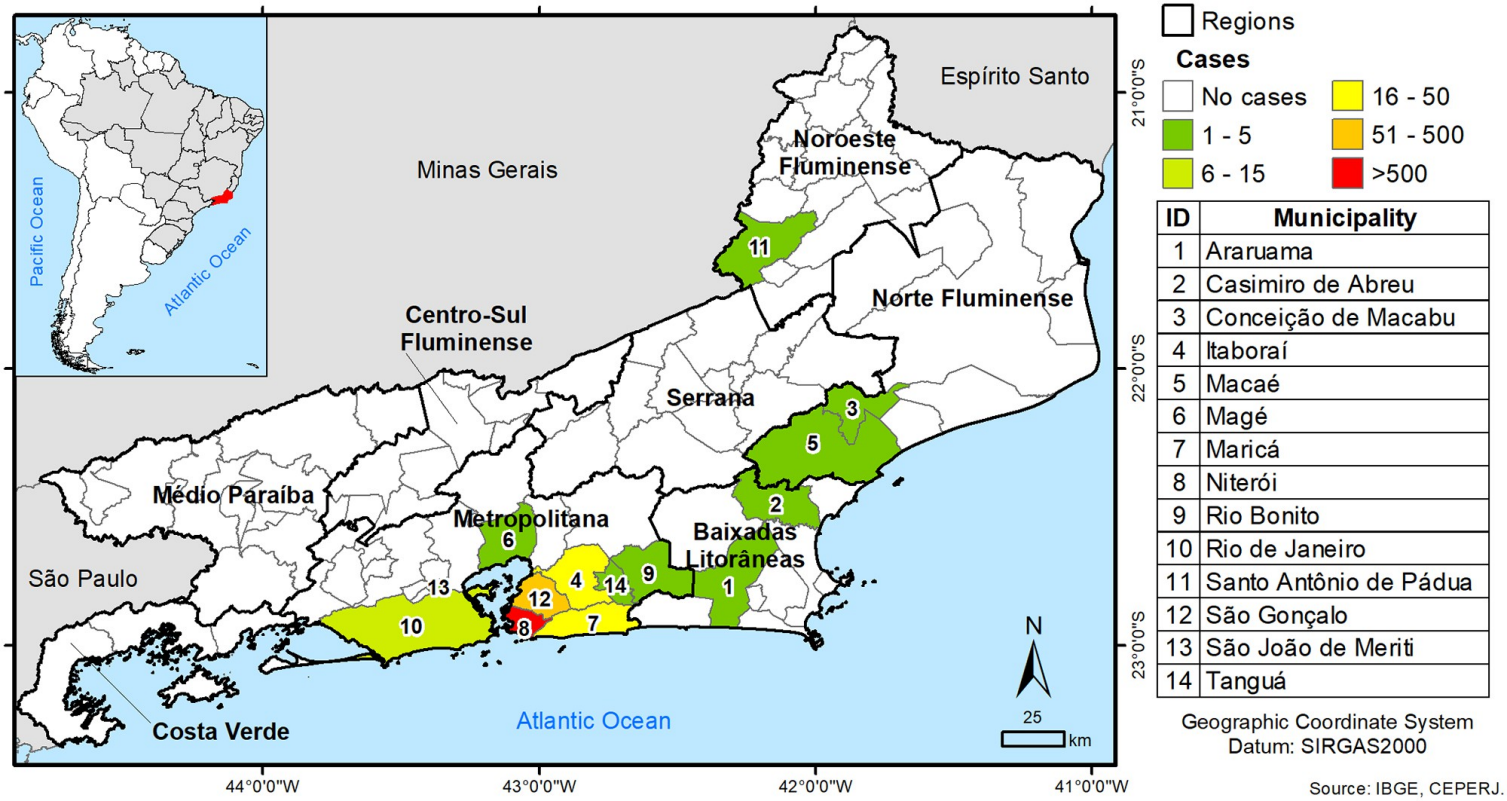
Rio de Janeiro state (study area), Regions and the municipalities from which patients with dermatophytosis (cases) originate (https://www.ibge.gov.br/en/geosciences/downloads-geosciences.html).

The majority of patients reside in municipalities belonging to the Metropolitana, Baixada Litorânea, Norte Fluminense, and Noroeste Fluminense Regions. Among municipalities, three were the most frequently referred: Niterói (n = 1,121), São Gonçalo (n = 267) and Maricá (n = 32), Metropolitana Region; (Fig 1).

**Fig 2:**
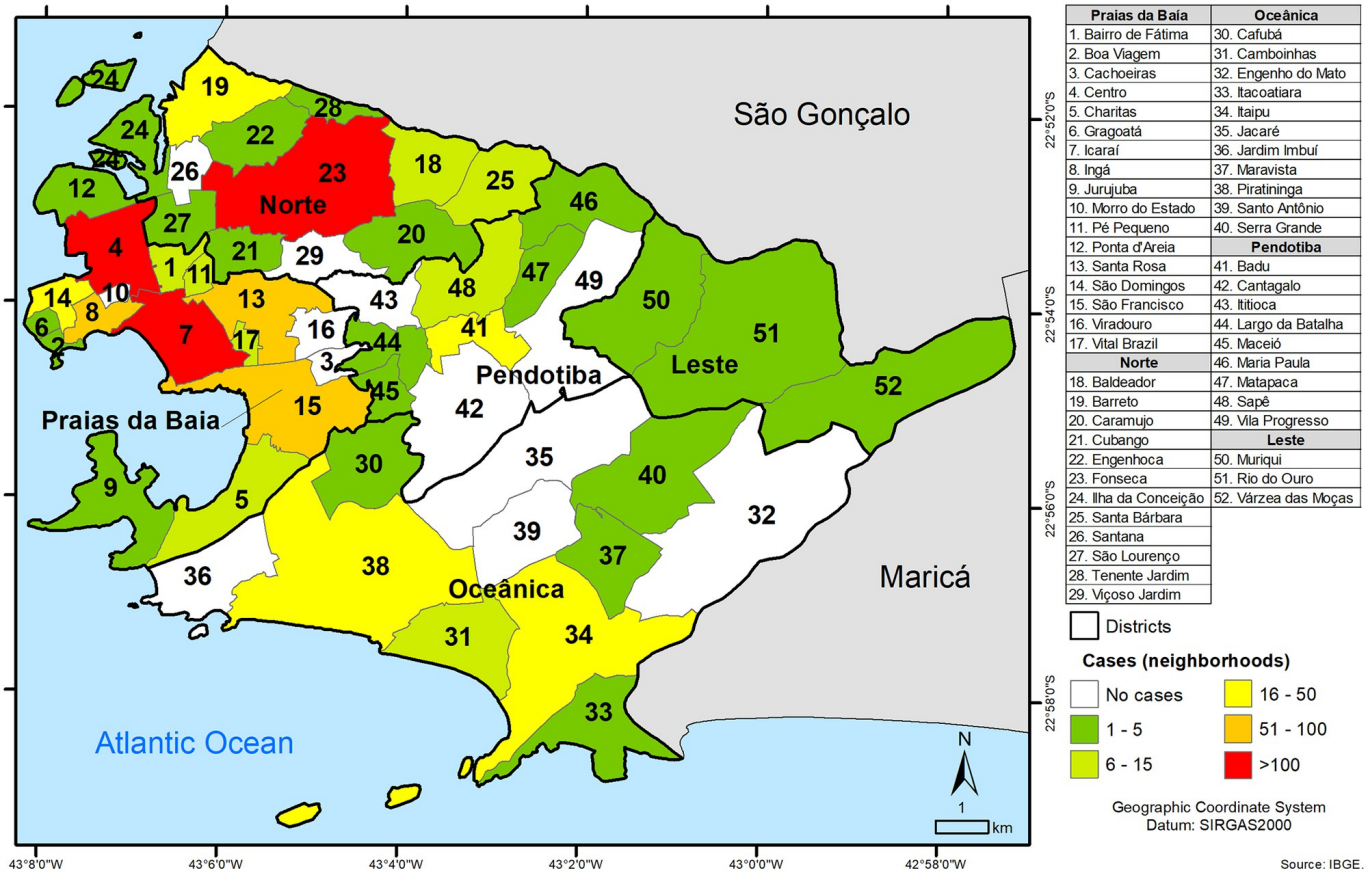
Districts and neighborhoods where patients with dermatophytosis from Niterói reside (https://www.ibge.gov.br/en/geosciences/downloads-geosciences.html).

In the municipality of Niterói, the largest number of cases was concentrated in Icaraí (n = 404) followed by Centro (n = 169) and Fonseca (n = 101) neighborhoods (Fig 2).

DME and/or culture isolation allowed the laboratorial confirmation of dermatophytosis in 1,485 individuals, that is, more than half of all individuals (54.5%) enrolled in this study. The annual distribution of these cases was homogeneous, with an average of 240.3 patients/year. A variation between 18 and 106 years old of the studied population was verified, (x = 58.9; σ ± 17.5 years), and women were more frequently affected (61.3%; x = 59.5; σ ± 17.8 years; p < 0.0001, Chi-square test). The mostly represented age group varied between 61 and 80 years (Table 1; p > 0.05, Chi-square Test). There was no preferential gender distribution (Table 1; p > 0.05, Chi-square Test).

**Table 1:** Distribution of dermatophytosis according to age and gender.

Age (years)	Gender		Total
	Male n (%)	Female n (%)	
18–39	96 (39.1)	150 (60.9)	246
40–60	198 (39.2)	307 (60.8)	505
61–80	235 (39.8)	355 (60.2)	590
≥81	45 (31.2)	99 (68.8)	144
Total	574 (38.7)	911 (61.3)	1485

*Tinea corporis* (31%), *Tinea unguium* (toe nail, 28%) and *Tinea pedis* (27%) were the most common dermatophytosis. Just over ten percent of all individuals (n = 170) had dermatophytosis in multiple anatomical sites (more than two body locations). Onychomycosis occurred in a similar frequency in the left and right halluces (n = 218, 31.1% and n = 233, 33.3%, respectively) while bilateral involvement was equally represented (n = 250; 35.6%; p> 0.05). Unilateral involvement (right or left halluces) was the most frequent onychomycosis location (p < 0.0001).

Skin scrapings represented the most frequent clinical specimen while nails (toenail or fingernail) contributed with more than half of them (n = 855; 57.6%). Hyaline septate hyphae (842/1485 patients) were detected by Direct Mycological Examination (DME), 16.05% of which arthroconidiate (n = 238), 24.6% (n = 366) contained only blastoconids, while a reduced percentage showed both micromorphological structures (2.62%, n = 39). Bacteria were detected in over 70% (n = 1045) of the samples. Mycological culture was positive for dermatophytes in 52.6% (n = 781) of all samples.

Patients were more frequently infected by *Trichophyton* spp. in the state of Rio de Janeiro (712/781), most of them *T*. *rubrum* (68.6%), followed by *T*. *mentagrophytes* (21.4%). *Microsporum canis*, *Nannizzia gypsea*, *Trichophyton tonsurans* and *Epidermophyton floccosum* showed reduced frequencies. In addition, bacteria detection in culture significantly reduced dermatophyte isolation (p<0.0001; McNemar test). Eighty-six individuals were infected/colonized by more than one species: *Trichophyton rubrum* and *Candida* spp. (29%), *T*. *rubrum* and *Fusarium* spp. (24.4%); *T*. *mentagrophytes* and *Fusarium (*15.1%); *T*. *mentagrophytes* and *Candida* spp. (11.6%), *T*. *rubrum* and *Neoscytalidium dimidiatum* (11.6%), among others. Table 2 summarizes the prevalence and distribution of dermatophytes and the anatomical site of the lesion.

**Table 2:** Absolute number and distribution of dermatophytes species according to the anatomical site of the lesion.

Sample	Dermatophytes						Mixed*
	*E*. *flocossum*	*M*. *canis*	*N*. *gypsea*	*T*. *rubrum*	*T*. *mentagrophytes*	*T*. *tonsurans*	
**Skin** n = 310	3	21	11	198	73	4	18
**Ungueal** n = 419	-	28	13	291	82	5	52
**Skin** **/Ungueal** n = 52	1	3	-	36	12	-	2
**Total** (n = 781)	4	52	24	525	167	9	72

* *Candida* spp., *Neoscytalidium dimidiatum*, *Fusarium* spp.

There was no dermatophyte preferential distribution between genders (Table 3). However, *M*. *canis* and *N*. *gypsea* were more frequently isolated in patients whose ages varied from 40 and 60 years old, while *T*. *rubrum* highest frequency was detected in younger patients (18 and 39 years-old; Table 4).

**Table 3:** Gender distribution of dermatophyte species isolated from the 781 diseased patients.

Dermatophytes	Gender		Total	*p*
	Male	Female		
*E*. *floccosum*	3(0.95%)	4(0.85%)	7	0.807*
*M*. *canis*	23 (7.32%)	29 (6.2%)	52	0.640
*N*. *gypsea*	11(3.5%)	13(2.78%)	24	0.719
*T*. *rubrum*	213 (67.8%)	316(67.7%)	529	0.977
*T*. *mentagrophytes*	66 (21%)	100(21.4%)	166	0.965
*T*. *tonsurans*	4(1.27%)	5 (1.1%)	9	0.935*
*Total*	314(100%)	467(100%)	781	

*Fischer Exact Test

**Table 4:** Distribution of dermatophytes according to the age group of the diseased investigated population.

Age Group/ Dermatophytes	18–39	40–60	61–80	≥81	Total	*p*
*E*. *flocossum*	3	4	nd	nd	7	-
*M*. *canis*	11	13*	26	2	52	<0.0001
*N*. *gypsea*	5	4*	12	3	24	<0.0001^ϯ^
*T*. *rubrum*	84*	194	186	59	523	<0.0001
*T*. *mentagrophytes*	35	51	67	13	166	-
*T*. *tonsurans*	1	2	4	2	9	-
**Total**	139	268	295	79	781	

* (p < 0.05)

^Ϯ^Fischer Exact Test

nd = not detected

### 3.1. Spatial Distribution of Dermatophytosis in State of Rio de Janeiro

**Fig 3:**
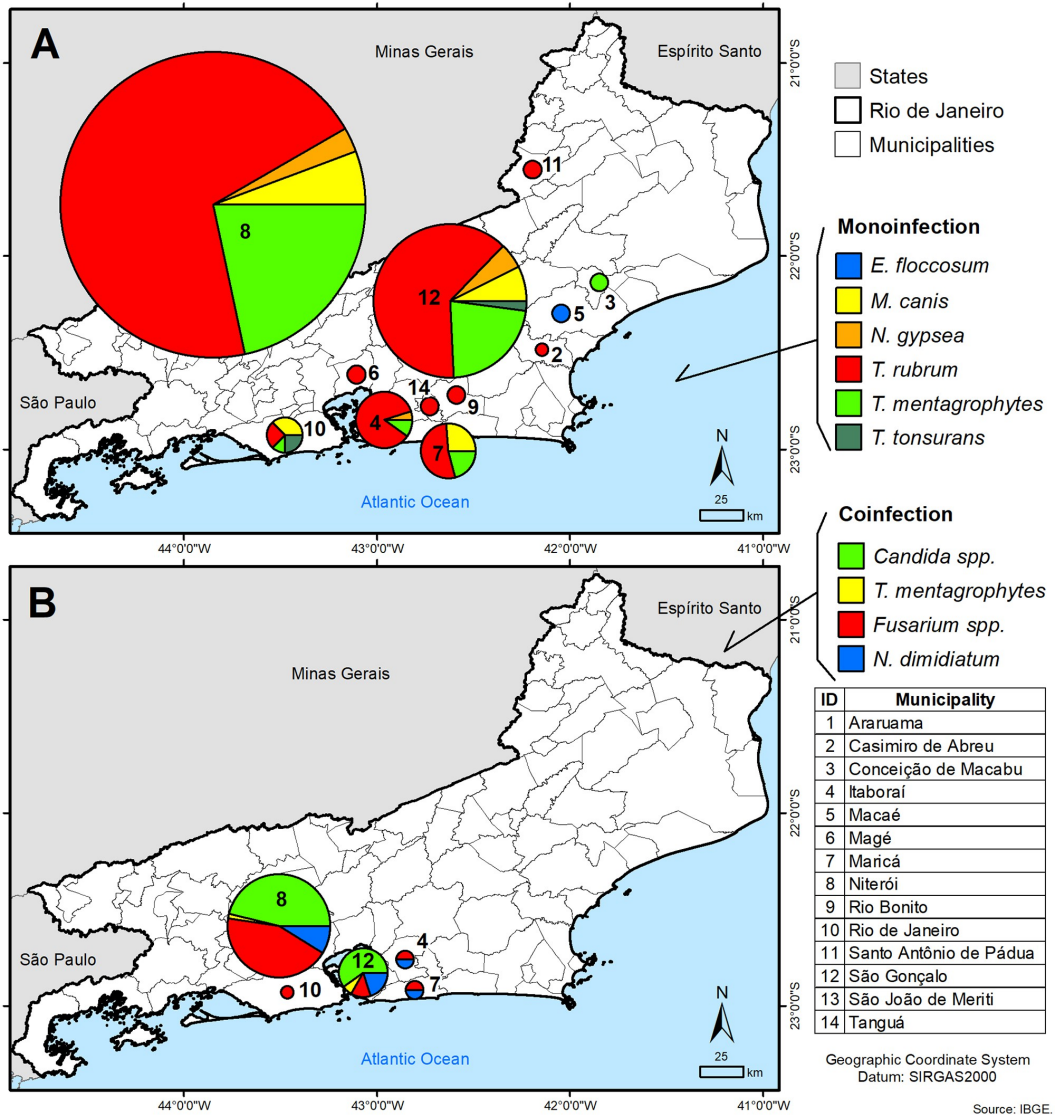
Spatial distribution of cases of dermatophytosis as: (A) monoinfection and (B) coinfection in Rio de Janeiro state municipalities (https://www.ibge.gov.br/en/geosciences/downloads-geosciences.html).

The single infections spatial case distribution in the studied area is represented in Fig 3A. All species presented homogeneous distribution while *T*. *tonsurans* appears to be restricted to the Rio de Janeiro capital and *E*. *floccosum* to the municipality of Macaé, located 190 kilometers northeast of the state capital. Fig 3B shows the distribution of patients from which mixed-infections (*Candida* spp., *Trichophyton mentagrophytes*, *Fusarium* spp. and *Neoscytalidium dimidiatum*) were detected, all concentrated in the Metropolitana region.

**Fig 4:**
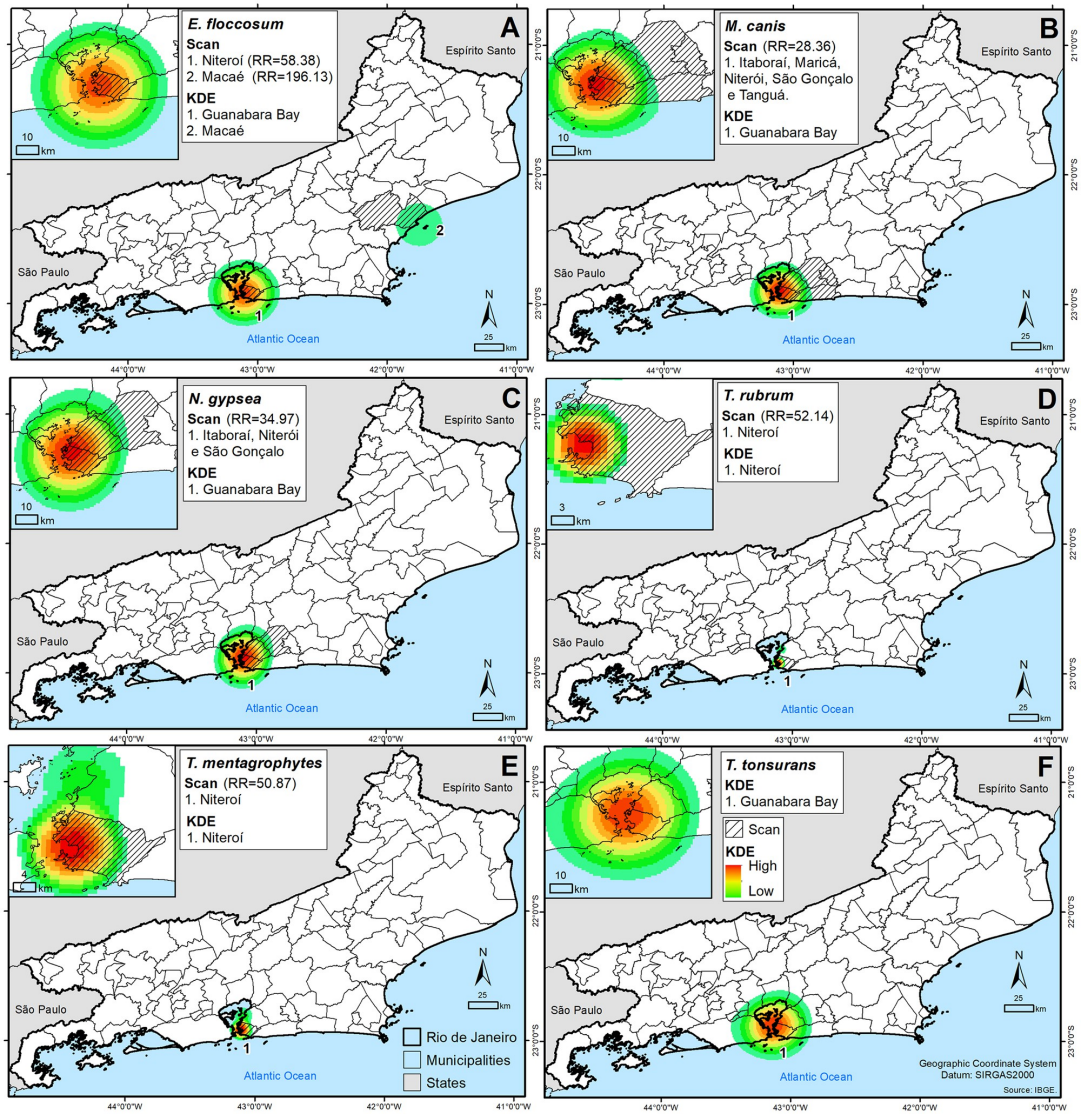
Application of EDK and Scan in monoinfections (A) *Epidermophyton floccosum*, (B) *Microsporum canis*, (C) *Nannizzia gypsea*, (D) *Trichophyton rubrum*, (E) *Trichophyton mentagrophytes* and (F) *Trichophyton tonsurans* in Rio de Janeiro state. (https://www.ibge.gov.br/en/geosciences/downloads-geosciences.html).

The KDE on dermatophytosis cases in Rio de Janeiro state (Fig 4) identified spatial clusters of cases with high density in Guanabara Bay (*E*. *floccosum*, *M*. *canis*, *N*. *gypsea*, *T*. *tonsurans*) and Niteroí (*T*. *rubrum*, *T*. *mentagrophytes*) but low density in Macaé (*E*. *floccosum*).

There was also a presence of significant spatiotemporal clusters (p-value ≤ 0.05) obtained by the Scan analysis. The Scan on dermatophytosis cases by *E*. *floccosum* (Fig 4A) identified two spatial clusters in Niterói (Relative risk (RR) = 58.38; Time frame (TF) = 2015–2017) and Macaé (RR = 196.13; TF = 2016). The Scan applied in *M*. *canis* cases (Fig 4B) identified one spatial cluster in the municipalities of Itaboraí, Maricá, Niterói, São Gonçalo and Tanguá (RR = 28.36; TF = 2014–2016). The Scan applied in *N*. *gypsea* cases (Fig 4C) identified one spatial cluster in the municipalities of Itaboraí, Niterói and São Gonçalo (RR = 34.97; TF = 2016–2018). The Scan applied in *T*. *rubrum* cases (Fig 4D) identified one spatial cluster in Niterói (RR = 52.14; TF = 2015–2017). The Scan applied in *T*. *mentagrophytes* cases (Fig 4E) identified one spatial cluster in Niterói (RR = 50.87; TF = 2015–2017). The Scan applied in *T*. *tonsurans* cases (Fig 4F) identified one non-significant spatial cluster in Niterói and São Gonçalo.

### 3.2. Spatial distribution of dermatophytosis in Niteróí/RJ

**Fig 5:**
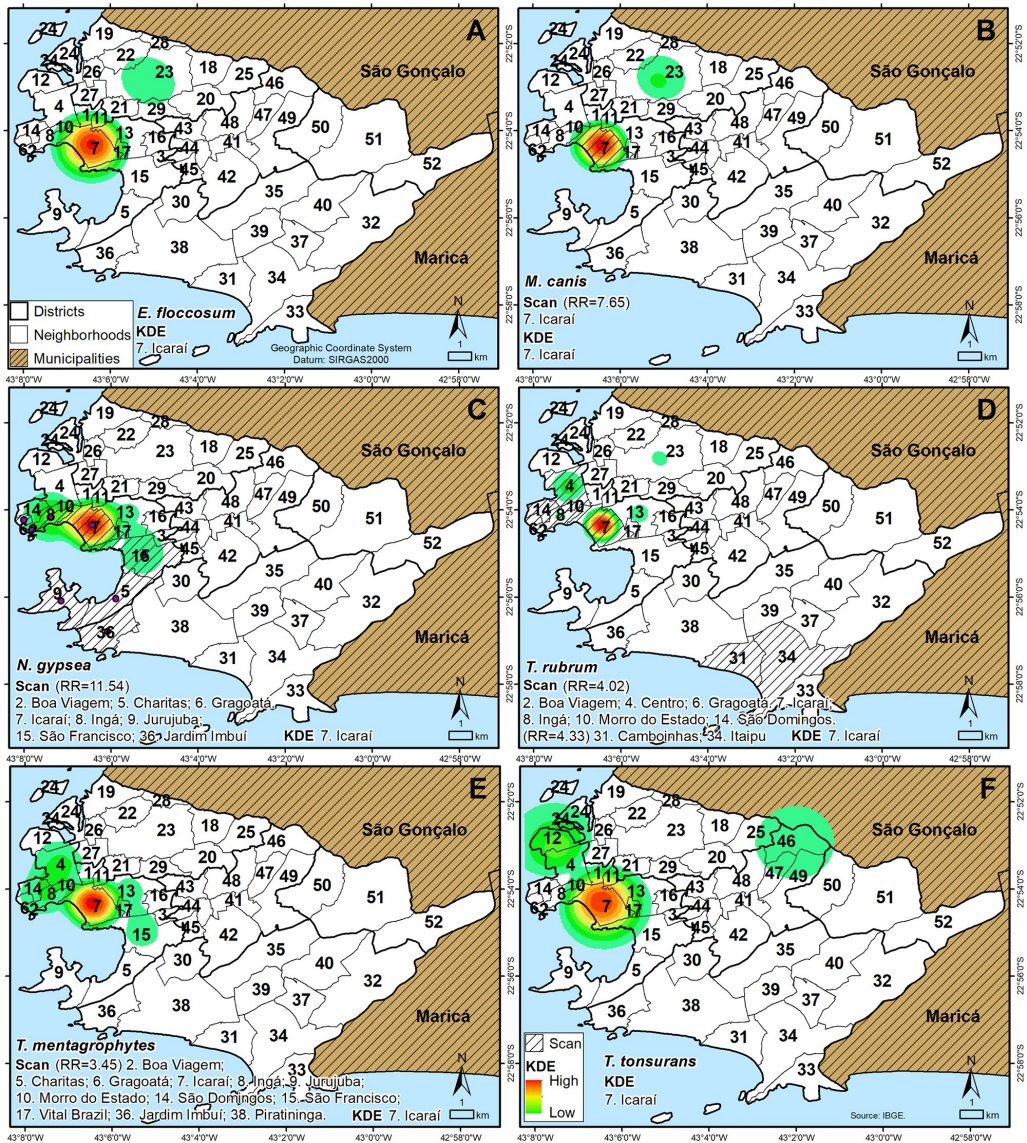
Application of EDK and Scan in monoinfections (A) *Epidermophyton floccosum*, (B) *Microsporum canis*, (C) *Nannizzia gypsea*, (D) *Trichophyton rubrum*, (E) *Trichophyton mentagrophytes* and (F) *Trichophyton tonsurans* in Niterói/RJ. (https://www.ibge.gov.br/en/geosciences/downloads-geosciences.html).

The KDE on dermatophytosis cases in Niterói/RJ (Fig 5) identified spatial clusters of cases with high density in the neighborhoods of Icaraí for all species (*E*. *floccosum*, *M*. *canis*, *N*. *gypsea*, *T*. *tonsurans*, *T*. *rubrum*, *T*. *mentagrophytes*).

The Scan analysis showed significant spatiotemporal clusters to: *M*. *canis* cases in Icaraí with RR = 7.65, TF = 2016–2018 (Fig 5B); *N*. *gypsea* cases in Boa Viagem, Charitas, Gragoatá, Icaraí, Ingá, Jurujuba, São Francisco and Jardim Imbuí with RR = 11.54, TF = 2018–2019 (Fig 5C); *T*. *rubrum* cases in Boa Viagem, Centro, Gragoatá, Icaraí, Ingá, Morro do Estado, São Domingos with RR = 4.02, TF = 2015–2017, Camboinhas and Itaipu with RR = 4.33, TF = 2017–2019 (Fig 5D); *T*. *mentagrophytes* cases in Boa Viagem, Charitas, Gragoatá, Icaraí, Ingá, Jurujuba, Morro do Estado, São Domingos, São Francisco, Vital Brazil, Jardim Imbuí and Piratininga with RR = 3.45, TF = 2016–2018 (Fig 5E). *E*. *floccosum* (Fig 5A) and *T*. *tonsurans* (Fig 5F) Scan analysis did not point significant spatial clustered cases.

**Fig 6:**
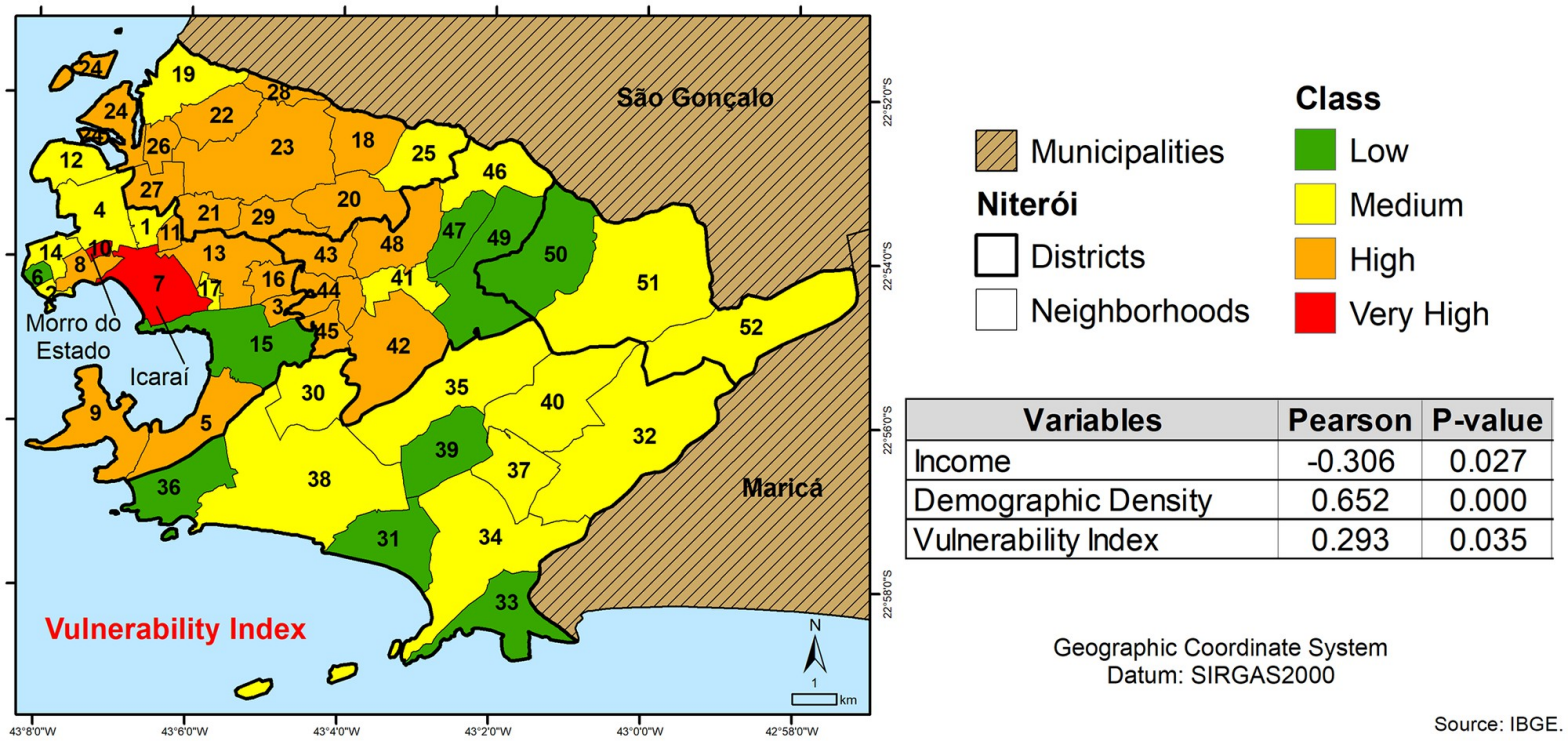
Vulnerability index map, showing the distribution of neighborhoods in the municipality of Niterói. In red are highlighted the neighborhoods of Icaraí (7) and Morro do Estado (10) (https://www.ibge.gov.br/en/geosciences/downloads-geosciences.html).

The Fig 6 shows the vulnerability index distributed according to neighborhoods in the municipality of Niterói, highlighting (red) the neighborhoods of Icaraí (7) and Morro do Estado (10) as very high classes. The Pearson’s correlation coefficient (r) was statistically significant (p-value ≤ 0.05), demonstrating that Vulnerability Index (r = 0.293) and Demographic Density (r = 0.652) were direct related with dermatophytosis cases whereas Income (r = -0.306) was inversely correlated.

## 4. Discussion

Although dermatomycosis are caused by yeasts of the genera *Candida*, *Malassezia*, *Trichosporon*, and some filamentous fungi such as *Fusarium* and *Aspergillus* [23], the main etiological agents involved in mycotic infections of the dermis are dermatophytes [24]. These constitute a specialized group of keratinophilic filamentous fungi that can affect the nails, hair, and skin of humans and, some of them, animals which cause a worldwide common cutaneous mycosis [25]. Nevertheless, dermatophytosis is usually overlooked and investments for broad research on controlling and treatment are scarce. This scenario is aggravated by the lack of compulsory notification and, although its low mortality rates, cutaneous lesions can present a chronic course and difficult treatment, mainly in immunocompromised patients [12,26].

In Brazil, dermatophytosis is still an unknown disease. Rio de Janeiro, Southeast Brazil, is a tropical climate state, the third most populated (around 17,000,000 inhabitants) and the second largest economy of the country. In addition, it is a major touristic center and annually attracts thousands of tourists from all over the world [27]. In the present study a total of 2,724 patients with suspicious cutaneous lesions were screened and over half of the biological samples provided the detection/isolation of dermatophytes (54.5%). Four other research groups reported similar frequencies: a population residing in Santa Catarina, South region [28], two studies in São Paulo state, Southeast region [29,30] and another one from the Northeast region [24]. On the other hand, these results are not in agreement with previous findings obtained by mycology services of four distinct Brazilian states: Paraná and Rio Grande do Sul, South region; Goiás, Central-West region, and Ceará, Northeast region, where percentages of 12.21%, 40%, 22.8% and 23.2% of these fungal infections were reported, respectively [12,13,31,32]. The pooled frequency of dermatophytosis in Brazil is lower (25%) than the one reported by the present study, according to a recent review by de Oliveira Pereira et al. (2021) [15]. The Human Mycoses Committee of the Japanese Society of Clinical Mycology revealed that among almost 7,000 suspected cases, 85.2% were positive for dermatophytosis [33] while a low frequency (0.98%) was reported in almost 3,500 students in Egypt [34]. This wide variation is in accordance with the great epidemiological variability already reported in different regions of the world and also within the same country [3].

As expected, the great majority of patients reside in the densely populated municipality of Niterói, in Icaraí neighborhood (Fig 2), the city’s most populated [18]. The patient’s mean age was 58.9 years, with a predominance of female, in accordance with previous studies by Ribeiro et al. (2015) [35], Sanguino et al. (2019) [13] and de Oliveira Pereira et al. (2021) [15], possibly explained by the everyday life habits, such as wearing shoes and excessive hand washing. Another possibility is related to the fact that women more frequently search for dermatological care. The average age as well as the most affected age group described in the Rio de Janeiro population, point to dermatophytosis as a condition more frequently observed in elderly, as previously described [15]. Indeed, dermatophytosis susceptibility in individuals over 65 years of age may correlate with therapeutic response to drugs and comorbidities common to this age group (peripheral vascular disease, diabetes, immunosuppression and physical trauma), in addition to nail anomalies [36]. *Tinea pedis* along with simultaneous *Tinea unguium*, the most common cutaneous mycosis in developing countries [37], were also described in distinct Brazilian populations [15,38], related to the frequent use of closed shoes and sports practice along with inadequate feet hygiene [32,38].

It is well known that countries located in warm tropical and subtropical climates are likely to present high frequencies of dermatophytosis and that epidemiological and geographical factors influence species distribution [39]. In such scenario, human miscegenation and/or migration can modulate dermatophyte species distribution [40]. Brazil is a country of continental dimension whose highly admixed population results from different ethnic migration throughout its history, unequally distributed within the different states. In addition, distinguishing dermatophyte species accurately is crucial since ecological grouping of origin (geophilic, zoophilic or anthropophilic) provides clues about the source of infection, ultimately contributing to avoid reinfection and also to the establishment of specific prevention measures. Lastly, dermatophytes origin also implicates in clinical presentation of the disease due to distinct host interaction [3,41].

In Rio de Janeiro, dermatophytosis was most commonly caused by *Trichophyton rubrum*, followed by *T*. *mentagrophytes*, *T*. *tonsurans*, *Microsporum canis*, *Nannizzia gypsea and Epidermophyton floccosum*. Our findings are in agreement with most of the research carried out in Brazil [15,32] and in the world [26], but differed from the studies of researchers from Pará state (North Brazil), where *T*. *mentagrophytes* predominates [42]. Nevertheless, in the present investigation the possibility of species misidentification cannot be ruled out, a limitation we acknowledge. This is because in a private laboratory diagnostic routine a more accurate level of species differentiation is not achieved. As an example, in 2017, Brito-Santos and collaborators [43] reported the first two cases of *Tinea capitis* by *Microsporum audouinii* in South America (Rio de Janeiro, Brazil), describing that both strains presented with poor growth compared to *M*. *canis*. In Brazil, the secondary role of zoophilic species was previously explained by a weak association between dermatophytosis and the cohabitation of animals and humans in urban areas [12]. As a matter of fact, a previous study in the Brazilian South region described a significant decline in the isolation of *M*. *canis* as a result of systematic veterinary control [44]. In the same way, a possibility to the reduced zoophilic dermatophyte frequency in Rio de Janeiro may be related to the three-decade sporotrichosis hyperendemics in Rio de Janeiro [45,46] which may have driven tutors to frequently search for veterinarian dermatological care whenever a skin lesion was noticed.

In the present study, non-dermatophyte fungi were detected as mixed infection/colonization mainly with *Candida* spp. or *Fusarium* spp. causing *Tinea unguium*. As a matter of fact, in Rio de Janeiro, *Candida albicans*, *Scytalidium* spp., *Geotrichum candidum*, *Aspergillus* spp., *Trichosporon* spp. and *Scopulariopsis brevicaulis* were previously shown to cause toenail and fingernail onychomycosis [47,48]. Importantly, we described a reduction in the frequency of culture dermatophyte isolation when bacteria were reported in DME. Likewise, dermatophytes negative culture results should be carefully interpreted whenever bacteria are noticed in DME. Indeed, as recently reported by Azzam et al. (2020) [49], different bacteria species produce antifungal substances like pyocyanin and hydroxy phenazine by *Pseudomonas aeruginosa*.

The majority of all dermatophytosis studies on the distinct Brazilian state populations were unable to demonstrate relationships between infectious agent and/or age group or sex [15]. Although uneasy to explain, in the present study we describe a shift from anthropophilic to non-anthropophilic dermatophytosis etiology from the age group between 18 and 39 towards the one between 40 and 60 years of age. This observation can be due to labor, lifestyle and/or recreational activities which can be distinct among these age groups.

The main advantage of spatiotemporal analysis lies in its ability to rapidly and easily show areas with the highest concentration and shifts of cases, thereby contributing towards planning, monitoring, and surveillance of dermatophytosis. The Kernel estimator identified spatial clusters of cases of high intensity caused by the three groups of environmental dermatophyte sources: geophilic, zoophilic and anthropophilic in Guanabara Bay. Indeed, Guanabara Bay is the focus of a great amount of human transit either by airplane (Santos Dumont Airport), ferry-boat or terrestrial transportations (Presidente Costa e Silva Bridge), for both financial and leisure purposes. This patch of ocean connects distinct municipalities from the Metropolitana area and is also a mandatory passage for one of the largest touristic state regions—the Baixada Litorânea and its famous beaches, such as Búzios and Cabo Frio.

### 4.1. Niterói dermatophytosis cases spatiotemporal distribution

The high density of spatial clusters of all dermatophyte species in Icaraí isn’t surprising since this neighborhood is the most densely populated in Niterói [18]. Interestingly, clusters of *M*. *canis* in Icaraí and Fonseca may be related to the fact that these neighborhoods are known for their traditional touristic parks: Campo de São Bento and Horto Botânico. These are extensive green areas of 50,000 and 10,000 Km2, respectively, with diverse adult and infant recreational and cultural activities. Likewise, they provide close interaction between environment-animal-man, either through pet walks with their tutors or because, sadly, they are customary points of abandonment. The countless number of abandoned animals in both parks is considered by the Center for Zoonosis Control and Coordination of Epidemiological Surveillance of the municipality a serious problem to be faced.

*N*. *gypsea* case clusters concentrated in the Guanabara Bay beaches of Niterói, named “Caminho Niemeyer”, are expected since this fungus is the most frequent geophilic etiologic agent of dermatophytosis. Therefore, this finding points to the need of future research focusing on the environmental distribution of *N*. *gypsea* in these touristic areas. On the other hand, the distribution of *T*. *rubrum* cases reflects the anthropophilic nature of this species, predominating in the most populous neighborhood of Niterói, Icaraí. Additionally, a high concentration of cases was detected in three seaside touristic neighborhoods: Gragoatá (Praias da Baía Region), Camboinhas and Itaipu (Oceânica Region). Taken together, these data will be relevant for the improvement of the current policy of touristic expansion in this municipality. The prospective elaboration of specific actions to control recreational parks (*M*. *canis*), beach sand (*N*. *gypsea*) and person to person (*T*. *rubrum*) transmission will contribute to the reduction of cases acquired by outdoor recreational activities.

Since the pattern of dermatophyte distribution is also powerfully shaped by migration, the growth of tourism and economic changes, this observation is particularly relevant in Rio de Janeiro. This capital is a well-known major tourism center in Brazil, resulting in the constant incoming of national and international visitors, recently embodied by major events such as the 2014 Football World Cup and the 2016 Olympic Games. In the first one, almost 120.000 people visited Niteroi’s major touristic points [50]. This observation may be specially important to explain the significant spatiotemporal clusters detected in Niteroi’s distinct neighborhoods, beginning in 2015 until 2019.

Also, it is worth noting the cluster of *T*. *rubrum* cases in Morro do Estado, the densely populated slumber in Centro, bearing a high vulnerability index. As a matter of fact, this may be explained by the major shift of drug dealers to Niterói municipality, eleven years ago, especially to the Morro do Estado slumber. This is due to the fact that they found shelter in Niterói slumbers after escaping from the newly installed police anti-drug task forces in the Capital, by the Rio de Janeiro Department of Public Safety [51].

## Conclusions

A limitation we acknowledge is the fact that the present analysis is not fully representative of Rio de Janeiro state since a single private laboratory was the source of all data and the majority of all patients reside in the Metropolitan Region of this state. Furthermore, some limitations are inherent to this type of investigation: incomplete medical records (lack of information); long evaluation period (potential change in external validity).

Dermatophytes belong to distinct ecological niches (infection sources) and its interaction with the human host can cause clinically distinguishable infections. The present study describes dermatophytes spatio-temporal shifts after two major international events in Rio de Janeiro, Brazil. We then shed light to the pressing need for specific measures of dermatophytosis prevention and controlling under the scope of both socio-economical and traveler’s medicine variables, particularly in touristic tropical localities.

## Supporting information

S1 Fig Study area: Regions and states of Brazil, and the municipality of Niterói/RJ. (TIF)
