# Supplementary figures and images for: Spatio-temporal six-year retrospective study on dermatophytosis in Rio de Janeiro, Southeast Brazil: A tropical tourist locality tale
Source: PLoS Negl Trop Dis. 2023 Apr 3;17(4):e0010865. doi: 10.1371/journal.pntd.0010865 (PMC10101643; doi:10.1371/journal.pntd.0010865)

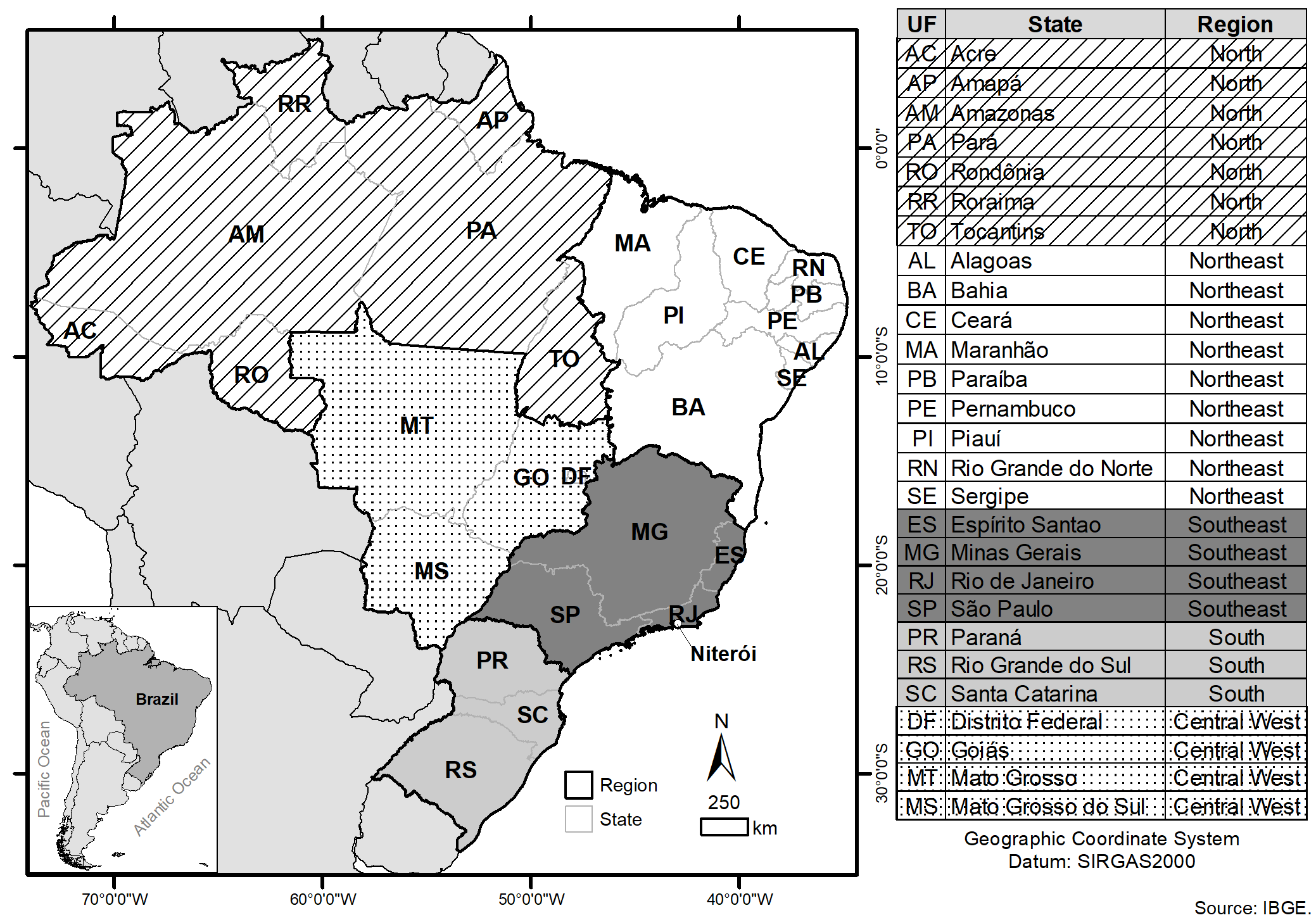

Supplement: S1 Fig — (TIF) [file pntd.0010865.s001.tif]
